# Release of Obstructive Prostatic Disease and Improvement of Erectile Dysfunction by Repetitive Prostatic Massage and Antimicrobial Therapy

**DOI:** 10.1100/tsw.2004.63

**Published:** 2004-10-20

**Authors:** Bradley R. Hennenfent, Antonio Novak Feliciano

**Affiliations:** The Prostatitis Foundation, The Dr. Antonio Novak Feliciano Clinic, USA

**Keywords:** benign prostatic hyperplasia, prostatitis, impotence, erectile dysfunction, prostatic massage, prostate, case report, obstruction, catheter

## Abstract

We report on a 69-year-old male who presented with an indwelling urinary catheter due to obstructive prostatic disease. The patient also complained of impotence. With repetitive prostatic massage combined with antimicrobial therapy the urinary obstruction was released, the patient's urinary symptoms resolved, and sexual function improved.

## INTRODUCTION

Either benign prostatic hyperplasia (BPH) or prostatitis can cause obstructive prostate disease. BPH arises in the transition zone of the prostate gland and slowly strangles urinary flow as men age. The exact etiology of BPH remains unknown. Benign prostatic hyperplasia is exceedingly common, with up to 25% of elderly men requiring a transurethral resection of the prostate (TURP).

There is evidence that BPH and prostatitis occur simultaneously. Kohen and Drach found the prostatitis lesion in 98% of patients with benign prostatic hypertrophy.[1] Bedalov et al. found an incidence of prostatitis in BPH of 90.3% and isolated bacteria from tissue removed at transurethral resection of the prostate (TURP) in 82.1% of patients.[2] Reidash et al found that more than 70% of TURP specimens showed clinical or microscopic signs of infection.[3] Mohanty and Jolly grew bacteria from 42% of 100 patients with BPH who had tissue removed at TURP.[4] Another study found that 21% of men with benign prostatic hyperplasia had infected prostate tissue removed at TURP.[5] Dan et al. found Chlamydia in 3 of 100 men undergoing TURP.[6] Indirect evidence that infection accompanies BPH is that many men suffer a febrile illness after TURP, thus the controversy about whether all patients should get prophylactic antibiotics for their TURP.[7,8,9,10]

It is possible that an overlooked component of BPH may be the obstruction and infection of prostatic ducts with resultant swelling and distention of prostatic acini.

## CASE REPORT

F. P. a 69-year-old male presented to the clinic with a urinary catheter in place. The patient gave a history of increasing frequency of urination over a few years (the patient could not be specific), with urinary obstruction requiring catheter insertion occurring six months previously. He also reported impotence (no erection) for the past few years. The patient reported that a urologic workup had been done and that a transurethral resection of the prostate (TURP) had been recommended for him.

The catheter was removed. On examination, the prostate was 2 + enlarged, non-tender smooth and boggy. A Gram's stain of the anterior urethra revealed 0 to 8 white blood cells (WBCs) per 1000 times magnification under oil immersion field (OIF). (For Gram's stain reports the medical technicians examine 20 fields). During prostate massage, the examining physician drained approximately 15 c.c.s of grayish opaque prostatic fluid with gelatinous worm-like material. A Gram's stain of the expressed prostatic secretions (EPS) revealed 1 to 7 WBCs/OIF. A urinalysis after massage revealed 2 plus protein and 6 to 12 WBCs/OIF.

No cultures were done as the patient declined them due to cost. The patient was treated empirically with metronidazole 2 grams p.o. as a one-time dose. Ofloxacin 400 mg p.o. twice a day and minocycline 100 mg p.o. bid were prescribed.

Two days later the patient reported freer urination, but still the presence of frequency and nocturia. Urethral Gram's stain revealed 0 WBCs. The second digital rectal examination (DRE) was done and the prostate was still slightly enlarged 1+ in size and 7.5 c.c.s of grayish opaque prostatic fluid was expressed by prostatic massage. Gram's stain of the EPS revealed 0 to 4 WBCs/OIF. The post massage urine revealed trace protein, and 8 to 20 WBCs/OIF. The same medications, ofloxacin and minocycline were continued.

Three days later, the patient reported further improvement of urination. He also reported having obtained 60% of a normal erection. Gram's stain of the anterior urethra again revealed 0 WBCs/OIF. Upon DRE the prostate was flat but boggy. Five to 16 drops of grayish prostatic fluid with precipitates were obtained from prostatic massage. Gram's stain of the EPS revealed 8 to 28 WBCs/OIF. The urinalysis was without WBCs. Ofloxacin and minocycline were continued.

Two days later, the patient reported normal urination. The prostate was flat and firm to palpitation. Five to 6 drops of gelatinous opaque prostatic fluid with precipitates were obtained. A Gram's stain of the EPS revealed 0 to 8 WBCs/OIF. The ofloxacin and minocycline were continued.

Two days later, the patient was asymptomatic. At the DRE the prostate was flat and firm. Four to 5 drops of gelatinous opaque prostatic fluid was obtained by massage. Gram's stain of the EPS revealed 8 to 25 WBCs/OIF. Ofloxacin was discontinued and replaced by ampicillin/sulbactam (Unasyn). It is the clinic's practice to switch one or both antibiotics if the pus cell count in the EPS increases after 7 days of therapy.

Three days later, the patient continued to report normal urination. He reported having a 70% erection. At DRE the prostate was flat and firm. Five to 6 drops of gelatinous opaque prostatic fluid with precipitates was expressed by massage. Gram's stain revealed 5 to 26 WBCs/OIF.

Minocycline and ampicillin/sulbactam were continued and erythromycin 500 mg. qid was added. Ketaconazole 2 tabs daily was started due to signs of penile candidiasis.

Two days later, the patient reported being asymptomatic. The DRE was unchanged, and Gram's stain of the EPS revealed 0 to 4 WBCs/OIF. The medications were continued. A table of all the patient's WBC counts shows a trend toward normalization in all instances (Table 1).

Two days later, the patient remained asymptomatic. The DRE was unchanged. Gram's stain of the EPS revealed 0 to 4 WBCs/OIF. The patient elected to discontinue treatment at this time because of improvement in symptoms and for financial reasons.

Telephone follow-up at 1 month and six months indicated that the patient was still free of urinary symptoms. At five-year follow up the patient reported normal urination with occasional nocturia. His improvement in erectile function remained at 60 to 70%.

**TABLE 1**
**The WBC counts in the specimens collected from the patient.**

| | urethra low | urethra high | EPS low | EPS high | UA low | UA high |
|---|---|---|---|---|---|---|
| Drainage #1 | 0 | 8 | 1 | 7 | 6 | 12 |
| Drainage #2 | 0 | 0 | 0 | 4 | 8 | 20 |
| Drainage #3 | 0 | 0 | 8 | 28 | 0 | 0 |
| Drainage #4 | | | 0 | 8 | | |
| Drainage #5 | | | 8 | 25 | | |
| Drainage #6 | | | 5 | 26 | | |
| Drainage #7 | | | 0 | 4 | | |
| Drainage #8 | | | 0 | 4 | | |

The urethra, EPS, and the urine were all looked at with Gram's stain. Each slide was examined for 20 fields and the lowest number of WBCs and the highest number of WBCs was recorded for each fluid.

A plot of the patient's EPS WBC counts (Figure 1) shows that the laboratory data confirms the clinical impression that obstructed and infected prostatic acini were opened up and drained over the 8 prostate massages. Since the EPS was reported as a range from low to high, the low and high WBC counts were both plotted and labeled as EPS low and EPS high. The EPS WBC count started low in each instance, rose, and then fell towards zero as treatment continued.

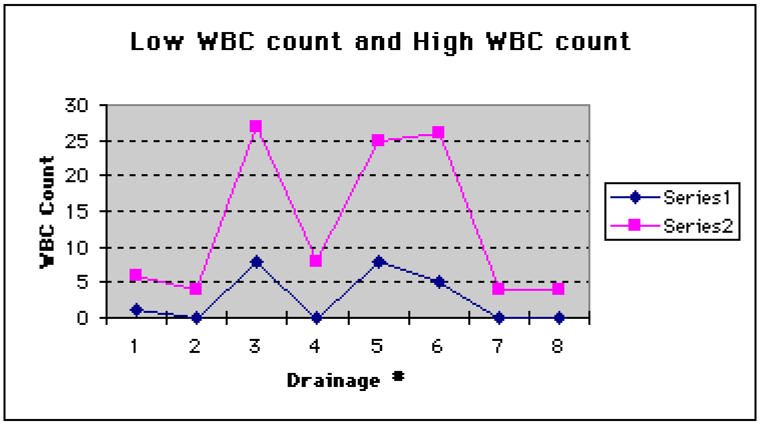

**FIGURE 1.** The rise and fall in the WBCs of the EPS at the eight different prostatic massages. Low and high values over 20 microscopic fields were recorded. In both cases the WBC count is seen to rise and then fall during therapy with repetitive massage and antibiotics, as if prostatic ducts were opened and drained of pus during therapy.

## DISCUSSION

Benign prostatic hyperplasia is exceedingly common with 90% of the elderly adult male population suffering from abnormal urination usually due to BPH.[11] The transurethral resection of the prostate (TURP) is considered the "gold standard" therapy for BPH and the TURP is the most common operation done for BPH.[12] There is controversy about the indications for doing a TURP, which is reflected in the geographical variation in the use of the TURP.[13] There is also a high rate of reoperation or retreatment after TURP.[14]

Medical therapy with alpha-andregenic blockers, and 5-alpha reductase inhibitors is playing a larger and larger role in the treatment of BPH. Variations of the TURP such as laser prostatectomy, transurethral needle oblation of the prostate (TUNA), and transurethral microwave thermotherapy (TUMT) of the prostate have been introduced and are being studied.

Interestingly, prior to these treatments the standard of care for prostatism, a term that encompasses both prostatitis and BPH, was prostatic massage. The authors, via computer searches and manual library searches have not found any evidence that prostatic massage has ever been studied in a scientific manner before it was summarily dropped as the standard of care for prostatism. In Medline, there are no randomized controlled trials on the clinical efficacy of prostatic massage for BPH or prostatitis.

The fact that infection may co-exist with BPH suggests that draining pus and infection from prostates might improve symptoms as was seen in this case report. This patient had obstructive prostatic disease diagnosed as BPH by his urologist, yet drainage of pus from the prostate alone relieved the obstructive symptoms.

## CONCLUSION

We present the case of a 69-year-old male presenting with urinary obstruction previously diagnosed as BPH necessitating a urinary catheter, and also complaining of impotence.

The patient was treated with 8 prostatic massages with copious drainage of purulent expressed prostatic secretions (EPS) and with antimicrobial therapy. Urinary symptoms resolved and improvement of erectile function occurred. Drainage of pus by repetitive prostatic massage combined with antibiotic therapy played a role in the therapy of this patient and avoided surgery for at least 5 years.
